# Effect of point-of-care echocardiography by noncardiologists on patient management in acute chest pain

**DOI:** 10.1007/s11739-025-04198-6

**Published:** 2025-11-24

**Authors:** Petr Grenar, Martin Jakl, Karel Mědílek, Jiří Nový, Jaromír Kočí, Jan Vaněk, Martina Čermáková, Jan M. Horáček, Radek Pudil

**Affiliations:** 1 Department of Military Internal Medicine and Military Hygiene, Military Faculty of Medicine, University of Defence, Trebesska 1575, 500 01 Hradec Kralove, Czech Republic; 2 Department of Emergency Medicine, University Hospital in Hradec Kralove, Hradec Kralove, Czech Republic; 3 First Department of Medicine—Cardioangiology, Charles University Prague, Medical Faculty and University Hospital Hradec Kralove, Prague, Czech Republic; 4 Centre of Advanced Technology, Faculty of Science, University of Hradec Kralove, Hradec Kralove, Czech Republic

**Keywords:** Point-of-care echocardiography, Acute chest pain, Education, Emergency medicine

## Abstract

As point-of-care echocardiography (POCE) has expanded beyond cardiology into general emergency medical practice, there has been a lack of standardized, evidence-based imaging protocols and training, thus leading to challenges with respect to the quality of cardiac imaging for acute cardiovascular syndromes. The ENDEMIC study aims to assess the effectiveness of POCE based on structured training for noncardiologists on the management of patients with acute chest pain in emergency departments. A total of 150 patients presenting with acute chest pain possibly of cardiovascular aetiology were enrolled in this prospective randomized clinical trial. Patients were assigned to either the POCE-assisted management group or the standard management group. Physicians performing POCE examinations received focused training based on the British Society of Echocardiography level 1 standard. The primary outcome was the length of stay in the emergency department (ED), and the secondary outcomes included the time to decision, the time to coronary angiography and diagnostic accuracy of the initial examination. Quality of acquired images was also evaluated. The results revealed that the POCE group had a significantly shorter length of ED stay (209.0 vs. 271.0 min, median difference 64 (31–97) min., p = 0.0003), time to decision (138.0 vs. 252.0 min, p < 0.0001) and time to coronary angiography (29.6 vs. 120.3 h, p = 0.027) than the control group. Furthermore, the error of initial diagnoses was lower in the POCE group (6.3% vs. 30.4%, RR = 0.21 [0.047–0.90]). The implementation of POCE by trained noncardiologists significantly reduced the duration of ED stay and enhanced the management of patients with acute chest pain in the ED. Systematic training enables physicians without prior echocardiography experience to perform POCE accurately and efficiently. Preregistered number of clinical trial: NCT05306730 registered 2022-04-01.

**Supplementary Information:**

The online version contains supplementary material available at https://doi.org/10.1007/s11739-025-04198-6.

## Introduction

In the past, echocardiography was performed exclusively by dedicated specialists. Today, physicians from a variety of specialties, including emergency medicine, intensive care, anaesthesiology and even primary care, perform bedside echocardiography. This shift has been driven by the advent of compact, easy-to-use ultrasound devices that have made it easier for non-specialists to perform cardiac imaging. Although point-of-care ultrasound has emerged over the past three decades, its growth may have accelerated since 2020, likely due to the COVID-19 pandemic [1–3].

**Fig. 1:**
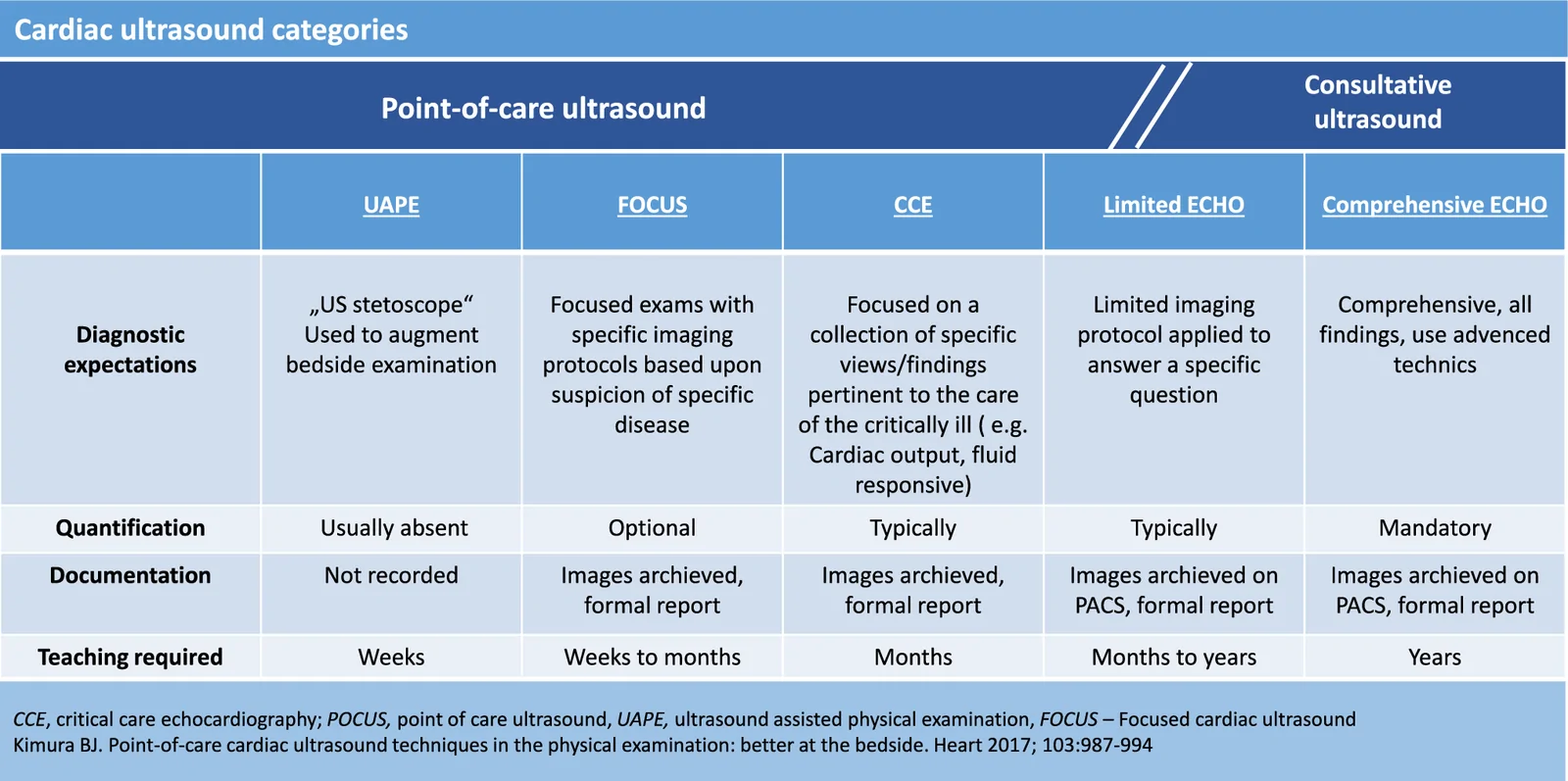
Definition of the levels of echocardiographic examination

Point-of-care echocardiography (POCE) is defined as the acquisition, interpretation and immediate clinical integration of echocardiographic images performed by clinicians at the patient’s bedside, rather than by a radiologist or cardiologist [4]. The different levels of cardiac ultrasound can be categorised according to the extent of the examination [5, 6] (Fig. 1). The current study corresponds to a detailed FOCUS examination according to the scheme.

One of the key uses of POCE is to diagnose patients with acute chest pain [7, 8]. Acute chest pain is one of the most common yet challenging presentations in emergency clinical practice, requiring immediate and accurate diagnosis and management. In the context of emergency medicine, where time is of the essence, POCE has emerged as a pivotal diagnostic tool beyond the traditional confines of cardiology departments. The use of POCE by trained non-cardiologists has begun to revolutionise patient assessment, providing a real-time view of cardiac function at the bedside. However, there is little evidence on the optimal scope of the examination and the training of the examiner. A key question remains: Can structured training in POCE for non-cardiologists significantly improve the speed and quality of patient care? And at what cost?

### Aims of the study

The study “Echocardiography by Non-cardiologist in Early Management of Patients With Chest Pain (ENDEMIC)” aims to assess the effect of controlled training of non-cardiologists on the use of POCE in the management of patients with acute chest pain. The primary objective was to assess the impact of POCE on the length of stay in the emergency department (ED). The primary safety endpoint was the occurrence of major adverse cardiovascular events (MACE) within 30 days of enrolment. Secondary objectives were to evaluate the impact of POCE on the time to decision, the time to coronary angiography and to assess the diagnostic accuracy of the initial examination. Quality of acquired images was also evaluated.

## Methods

We conducted a prospective, randomised cohort study at the Department of Emergency Medicine and First Department of Medicine—Cardioangiology, University Hospital Hradec Kralove, Czech Republic (ClinicalTrial.gov NCT05306730). The study protocol was approved by the Ethics Committee and was conducted in accordance with sthe Declaration of Helsinki. All patients provided informed written consent.

### Training on point-of-care echocardiography

The training programme was described in detail previously [9]. The training program enrolled four physicians who had not undergone systematic training in echocardiography (including self-education) and had performed examinations up to the level of ultrasound-augmented physical examination. The British Society of Echocardiography (BSE) Level 1 protocol [10] was chosen as the basis for determining the scope of the training. The following modalities were added to this training: diameter of ascending aorta, colour flow mapping of aortic and mitral valve in short axis projection and measurement of tricuspid valve gradient. This additional training was added to the original protocol to cover common and serious conditions encountered in the emergency department (e.g. aortic dissection, severe valvular disease and significant pulmonary hypertension).

The training programme consisted of several steps: an introductory course in focused echocardiography, a supervised examination of patients in whom at least 29 predefined findings had to be identified (Supplementum 1) and a final examination of real patient assessed by a specialist with European Association of Cardiovascular Imaging accreditation in transthoracic echocardiography. The total training period was approximately 6 months.

Physicians began performing POCE on study participants immediately after successfully completing the course. To maintain the quality of the examinations, graduates were required to document at least fifty examinations per year. In addition, once a year, graduates spent four hours in the EchoLab under the supervision of a mentor to verify their continuing competence in performing POCE.

### Selection of participants

Patients presenting to the ED with chest pain were eligible for the study. Inclusion criteria were as follows: 18 years of age or older; and pain that may have had a cardiovascular aetiology based on the patient’s history, physical examination, and ECG findings. Exclusion criteria were as follows: possible ST-elevation myocardial infarction (STEMI), active pacemaker, pregnancy, performance status of 4 on the Zubrod scale, refusal or inability to give informed consent and no suspicion of a cardiovascular aetiology for the patient’s symptoms after initial clinical examination (for example, a young individual with vertebral pathology presenting with acute, precisely localised, palpation-sensitive, and position-dependent pain, accompanied by a normal ECG).

Possible STEMI was defined according to current guidelines as new ST segment elevation of more than 1 mm in two or more anatomically contiguous leads (measured at the J point) in all leads except V2–V3, where the threshold is over 2 mm in men older than 40 years, over 2.5 mm in men younger than 40 years, and over 1.5 mm in women of any age[11]. Such patients were referred immediately for coronary angiography that could not be delayed by the ultrasound examination.

**Fig. 2:**
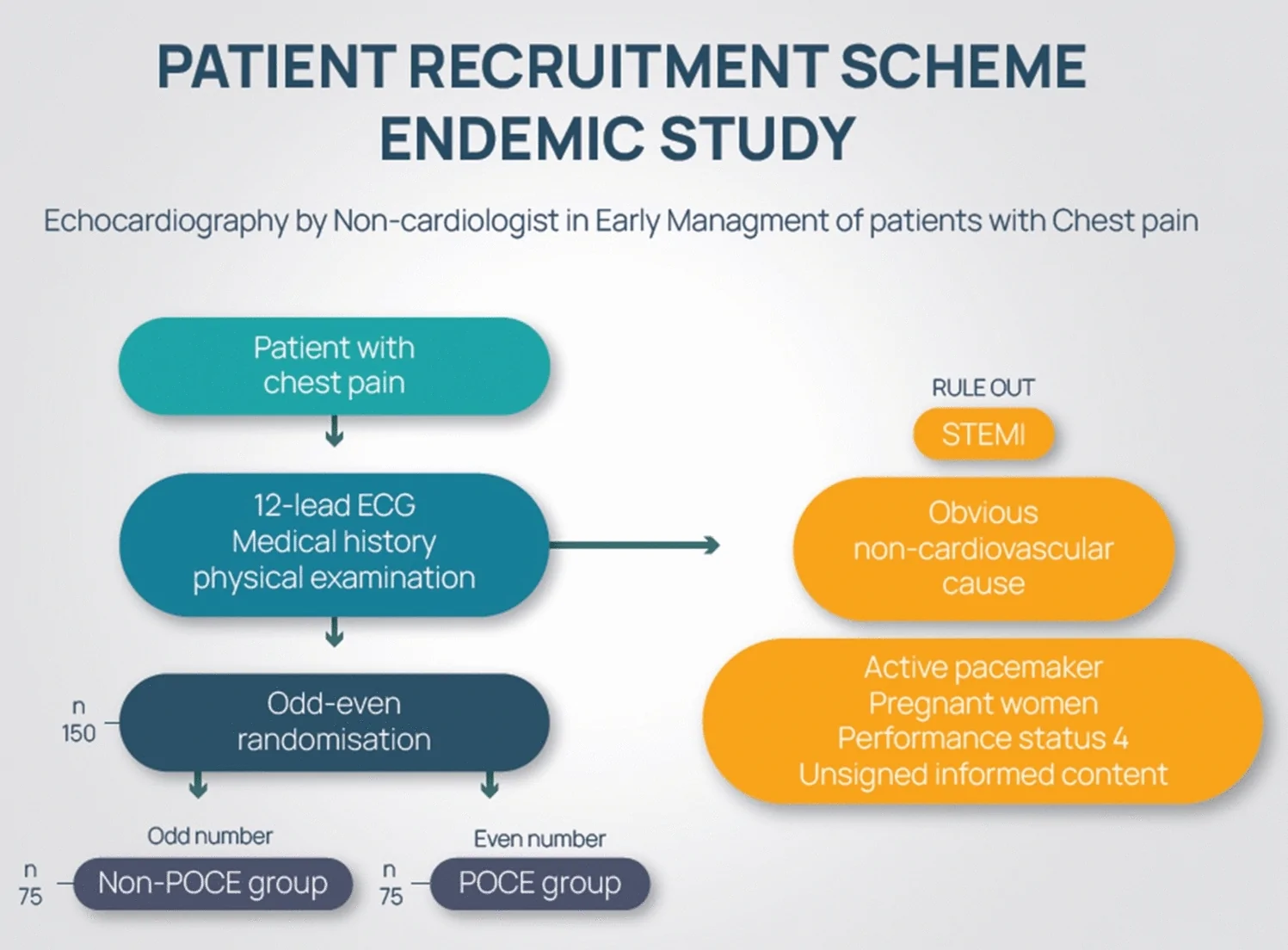
Patient recruitment scheme

Patients were not excluded on the basis of poor ultrasound visualisation. All patients were followed up for a minimum of 3 months. Patients were randomized in an even–odd manner to a POCE-assisted management group or a standard management group (Fig. 2).

### Echocardiography examination

POCE was performed following ECG and standard clinical examination, but no later than 90 min after arrival at the emergency department. It was performed by an operator who had completed the aforementioned training programme. The recommended scope of POCE was based on the BSE Level 1 protocol. However, the examining physician was authorised to extend the scope of the examination if necessary.

All images of the examination were archived in the hospital information system and reviewed post hoc by an EACVI (European Association of Cardiovascular Imaging) accredited echocardiographer and the approved supervisor of the British Society of Echocardiography to assess the accuracy of the examinations performed.

In patients in control group, echocardiography was performed only if required by specific ESC guidelines (in case of 3-h troponine protocol etc.). In that case, the examination was performed by a cardiology fellow.

### Outcomes

The primary endpoint of the study was the length of ED stay. The start of the ED stay was defined as the time of the patient’s first contact with the ED, regardless of whether the patient was referred by the emergency medical service or self-referred. The end of the ED stay was defined as the moment the patient physically departed the ED, either through hospital admission or discharge home.

The time to decision was defined as the time at which the final decision regarding diagnosis or the initiation of a guideline-based therapeutic intervention was made in the ED, depending on which occurred later.

Time to revascularisation and occurrence of MACE were obtained from the patients’ medical records and confirmed by evidence from the Czech National Population Registry.

MACE was defined as the composite endpoint of cardiovascular death, non-fatal myocardial infarction, or unplanned hospitalisation due to cardiovascular disease within 30 days of the clinical examination. Because of the absence of non-fatal myocardial infarction and very low incidence of death in discharged patients, discrepancy between working and final diagnosis was added as a secondary safety endpoint.

A working diagnosis was considered accurate if it shared both a pathophysiological mechanism and diagnostic criteria with the final diagnosis (e.g., a working diagnosis of acute coronary syndrome was considered accurate if the final diagnosis was myocardial infarction or unstable angina but not takotsubo cardiomyopathy). In case of discrepancy between the working and final diagnosis, the final diagnosis was determined by independent review.

### Ethics approval

This study was approved by the local ethical committee (Ethics Committee of University Hospital in Hradec Kralove, accredited by the Office for Human Research Protections under the number IORG0008813). The study was conducted in accordance with the Declaration of Helsinki. All patients provided written informed consent.

### Statistical analysis

Categorical variables are expressed as group counts and relative frequencies (%), and continuous variables are expressed as group means, standard deviations and totals (N). Effect size is expressed as median of difference (ΔM) with 95% confidence interval (95% CI).

As most of the continuous variables examined here did not follow a normal distribution (as indicated by exploratory analysis and the Shapiro–Wilk test), the non-parametric Wilcoxon rank-sum test was used to compare continuous outcomes between groups. Categorical variables were compared using Fisher’s exact test. Statistical significance was set at alpha = 0.05 for all tests. In cases of multiple testing of similar hypothesis (e.g. various ways of reducing management time), the Bonferroni-Holm correction for the nominal level of statistical significance was applied to keep the familywise Type I error rate alpha at 0.05. Statistical analyses were performed using R software (R version 3.5.3; R Foundation for Statistical Computing, Vienna, Austria).

In this study, we aimed to test the hypothesis that the implementation of POCE would lead to a reduction in the length of stay in the ED of at least 15% (≈ 30 min). We performed a power analysis to determine the appropriate sample size for our study. The desired power was set at β = 0.8. The effect size was estimated on the basis of preliminary data in which the length of stay in the ED was determined to be 176 ± 90 min. Taking these parameters into account, the sample size was determined to be 430 patients.

An interim analysis was performed after every 50 patients. If both the primary and secondary endpoints reached statistical significance (including the Bonferroni-Holm correction), the Data Safety Monitoring Board was asked to assess the ethics of continuing the study. Given the continued superiority of POCE in both reducing the length of stay in the ED and improving diagnostic accuracy, the Board recommended early termination of the study after enrolment of 150 patients.

## Results

### Characteristics of study subjects

Between September 2022 and October 2023, a total of 150 patients were enrolled in the study. The baseline demographic and clinical data of the patients are summarized in Table 1. The descriptive characteristics were well balanced across the groups.

**Table 1:** Comparison of baseline characteristics between the POCE and control groups

	POCE group (n = 75)	Control group (n = 75)	p
Age [years; mean ± SD]	61.1 ± 17.8	66.1 ± 15.6	0.072
Male sex [n (%)]	50 (66.7)	41 (54.7)	0.181
Caucasian ethnicity [n (%)]	75 (100)	75 (100)	1.0
Chronic coronary artery disease [n (%)]	19 (25.3)	20 (26.7)	1.0
Diabetes mellitus [n (%)]	19 (25.3)	12 (16.0)	0.226
Arterial hypertension [n (%)]	39 (52.0)	42 (56.0)	0.743
Severe valvular disease [n (%)]	4 (5.3)	4 (5.3)	1.0
Atrial fibrillation [n (%)]	7 (9.3)	10 (13.3)	0.608
Active smoker [n (%)]	14 (18.7)	14 (18.7)	1.0
Former smoker [n (%)]	15 (20.0)	17 (22.7)	0.690
Antiplatelet therapy [n (%)]	20 (26.6)	23 (30.7)	0.718
Anticoagulation therapy [n (%)]	8 (10.6)	13 (17.3)	0.239
GRACE score [points]	96.0 (69.0–125.5)	100.0 (64.5–134.0)	0.536
HEART score [points; median (IQR)]	5.0 (3.0–7.0)	5.0 (3.0–6.0)	0.648
Indication of further follow-up [n; %]	30 (40.0)	26 (24.7)	0.612
Patients requiring hospital admission [n; %]	25 (33.3)	14 (18.7)	0.062

*POCE* point-of-care echocardiography; *GRACE* Global Registry of Acute Coronary Events [1]; *HEART* History, ECG, Age, Risk factors and Troponin (18,665,203)[2]; *ECG* electrocardiography

In the POCE group, echocardiography was performed in all patients. All records were subsequently reviewed for quality of image acquired, and the real extent of the examination was recorded. Of the 16 projections based on the BSE Level 1 protocol, 12.5 ± 2.5 (78.4 ± 15.9%) were actually performed. An additional 5.4 ± 1.6 projections were performed on average. A total of 91.0% of the projections were fully conclusive, 7.6% were partly inconclusive, and 1.4% were completely inconclusive (Table 2). Regional wall motion abnormity (RWA), including septal D-shape, was present in 38.5% of patients; however, it was present in only 21.4% of patients with confirmed acute coronary syndrome.

**Table 2:** Extent of POCE examinations

Projection	Number patients with a projection performed [n (%)]
	fully conclusive [n (%)]
	partially inconclusive [n (%)]
Parasternal ascending aorta**	69 (92.0)
	67 (89.3)
	1 (1.3)
PLAX (2D)*	67 (89.3)
	61 (81.3)
	5 (6.7)
PLAX (LVIDd)*	66 (88.0)
	46 (61.3)
	14 (18.7)
PLAX with CFM aortic and mitral valve*	71 (94.7)
	58 (77.3)
	13 (17.3)
PSAX outflow (2D)*	46 (61.3)
	42 (54.7)
	4 (5.3)
PSAX outflow with CFM aortic valve**	54 (72.0)
	50 (66.7)
	3 (4.0)
PSAX outflow with CFM mitral valve**	34 (45.3)
	30 (40.0)
	2 (2.7)
PSAX base (2D)*	39 (52.0)
	38 (50.7)
	0 (0)
PSAX mid (2D)*	61 (81.3)
	61 (81.3)
	0 (0)
PSAX apex (2D)*	28 (37.3)
	28 (37.3)
	0 (0)
A4C (2D)*	71 (94.7)
	61 (81.3)
	10 (13.3)
A4C (M-Mode) TAPSE*	71 (94.7)
	71 (94.7)
	0 (0)
A4C with CFM tricuspid valve*	34 (45.3)
	34 (45.3)
	0 (0)
A4C Tricuspid gradient (CW)**	52 (69.3)
	52 (69.3)
	0 (0)
A4C with CFM mitral valve*	60 (80.0)
	56 (74.7)
	3 (4.0)
A5C (2D)*	18 (24.0)
	15 (20.0)
	3 (4.0)
A5C with CFM aortic valve*	28 (37.3)
	27 (36.0)
	1 (1.3)
A2C (2D)	68 (88.0)
	58 (77.3)
	8 (10.7)
A2C with FCM mitral valve	44 (58.7)
	40 (53.3)
	4 (5.3)
A3C	64 (85.3)
	58 (77.3)
	6 (8.0)
A3C with CFM aortic valve	38 (50.7)
	34 (45.3)
	4 (5.3)
Subcostal 4 C (2D)*	63 (84.0)
	49 (65.3)
	12 (16.0)
Subcostal IVC (2D)*	67 (88.0)
	64 (85.3)
	3 (4.0)
Subcostal IVC (M-Mode)*	58 (74.7)
	56 (74.7)
	1 (1.3)
Suprasternal long axis	3 (4.0)
	3 (4.0)
	0 (0)
Left ventricular systolic function assessed by at least two independent projections	74 (98.6)
Mitral valve assessed by at least one projection	62 (82.6)
Aortic valve assessed by at least one projection	62 (82.6)
IVC assessed by at least one projection	56 (74.6)

^*^ projections required by the BSE Level 1 protocol

^**^ additional projections involved in pre-study education

*POCE* point-of-care echocardiography; *2D* 2D dynamic image without CFM acquisition; *CFM* colour flow mapping; *PLAX* parasternal long axis projection; *LVIDd* Left ventricular internal diameter in diastole; *PSAX* parasternal short axis projection; *A4C* apical 4-chamber projection; *TAPSE* tricuspid annular plane systolic excursion; *A3C* apical 3-chamber projection; *A2C* apical 2-chamber projection; *IVC* inferior vena cava

### Main results

**Fig. 3:**
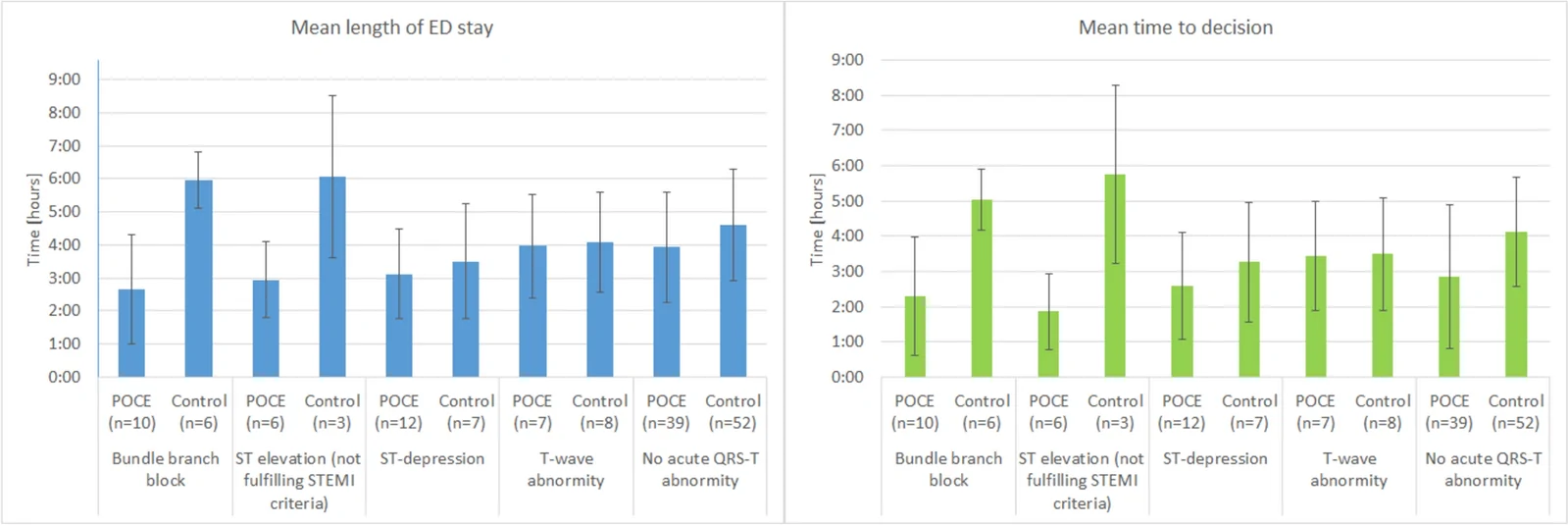
Reduction of management times depending on the initial ECG

**Fig. 4:**
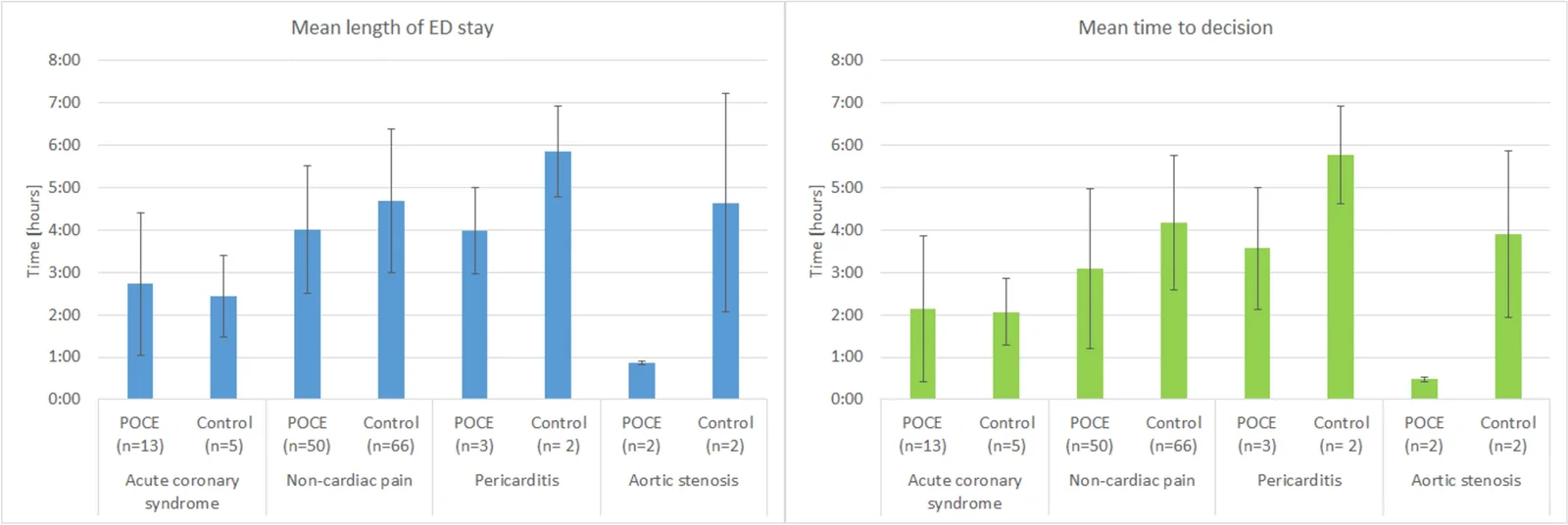
Reduction of management times depending on the final diagnosis

The use of POCE resulted in a significant shortening of ED stay, time to decision and time to coronary angiography (Table 3). The reduction in the time of ED stay depended on the ECG findings and final diagnosis, p = 0.019 and p = 0.047, respectively (Fig. 3 and Fig. 4; Supplementum 2 and 3). However, the small number of patients in some subgroups meant that meaningful subgroup analysis was not possible.

**Table 3:** Comparison of the POCE and control groups

	POCE group (n = 75)	Control group (n = 75)	p
Time to decision in whole study group [min; mean (IQR)]	138.0 (68.5 − 230.5)	252.0 (165.5 − 304.0)	< 0.0001
Duration of ED stay in whole study group [min; mean (IQR)]	209.0 (143.5 − 260.0)	271.0 (206.5 − 336.0)	0.0003
Time to decision among hospitalized patients [min; mean (IQR)]	112.0 (51.0 − 180.0)	183.5 122.75 − 252.0	0.024
Duration of ED stay among hospitalized patients [min; mean (IQR)]	178.0 (75.0 − 224.0)	219.5 (145.5 − 293.75)	0.058
Time to coronary angiography [hours]	29.6 (23.6–38.1)	120.3 (45.6–147.9)	0.027
MACE at day 30 [n; %]	0 (0)	0 (0)	1.0
All cause deaths at day 90 [n; %]	1 (1.33)	1 (1.33)	1.0

*POCE* point-of-care echocardiography; *ED* emergency department; *IQR* interquartile range; *MACE* Mayor cardiovascular adverse events

The incidence of adverse events was lower than expected; therefore, the study would be underpowered for a reliable analysis even if enrolment were not stopped. We found no evidence to support the hypothesis that the use of POCE increases the risk of an incorrect working diagnosis (6.3% in the POCE group vs. 30.4% in the control group, p = 0.997 for inferiority). Although this was not a prespecified endpoint, the risk ratio suggests that the use of POCE resulted in superior diagnostic accuracy: RR = 0.21 (0.047–0.90).

## Discussion

The present study stands at the intersection of educational strategy and clinical effectiveness and provides insight into how systematic training in echocardiography can equip non-cardiologists with the skills needed to effectively manage patients with acute chest pain. We have shown that physicians with no previous experience can be effectively trained to perform POCE, and that their clinical performance resulted in shorter emergency department stays and more accurate diagnoses.

Despite the widespread use of echocardiography in patients with acute cardiovascular disease, recommendations for POCE in the emergency setting remain vague, particularly regarding the optimal extent and timing of examinations [7, 8, 12–14]. This lack of clarity is a cause for concern and is in line with the recognition of POCE as a major health technology risk [14]. In addition, evidence on the extent of necessary education is limited and primarily based on national standards, despite calls for high-quality, competency-based cardiac imaging service delivery [15].

The efficacy and accuracy of POCE has been evaluated in several previous studies. These studies have shown acceptable agreement between POCE and comprehensive echocardiography, as well as increased diagnostic confidence among physicians, although without significant clinical benefit [16–18]. Recent research has demonstrated reductions in patient management time but not mortality endpoints, but there is no detailed information on examiner training and scope of examination [19, 20]. However, we believe that it is not echocardiography itself that should be the focus of scientific interest, but rather the way in which it is taught, indicated and performed. In particular, it needs to be shown that POCE can be beneficial to ordinary emergency physicians, not just highly experienced specialists. This is where we see the greatest contribution of this study.

**Fig. 5:**
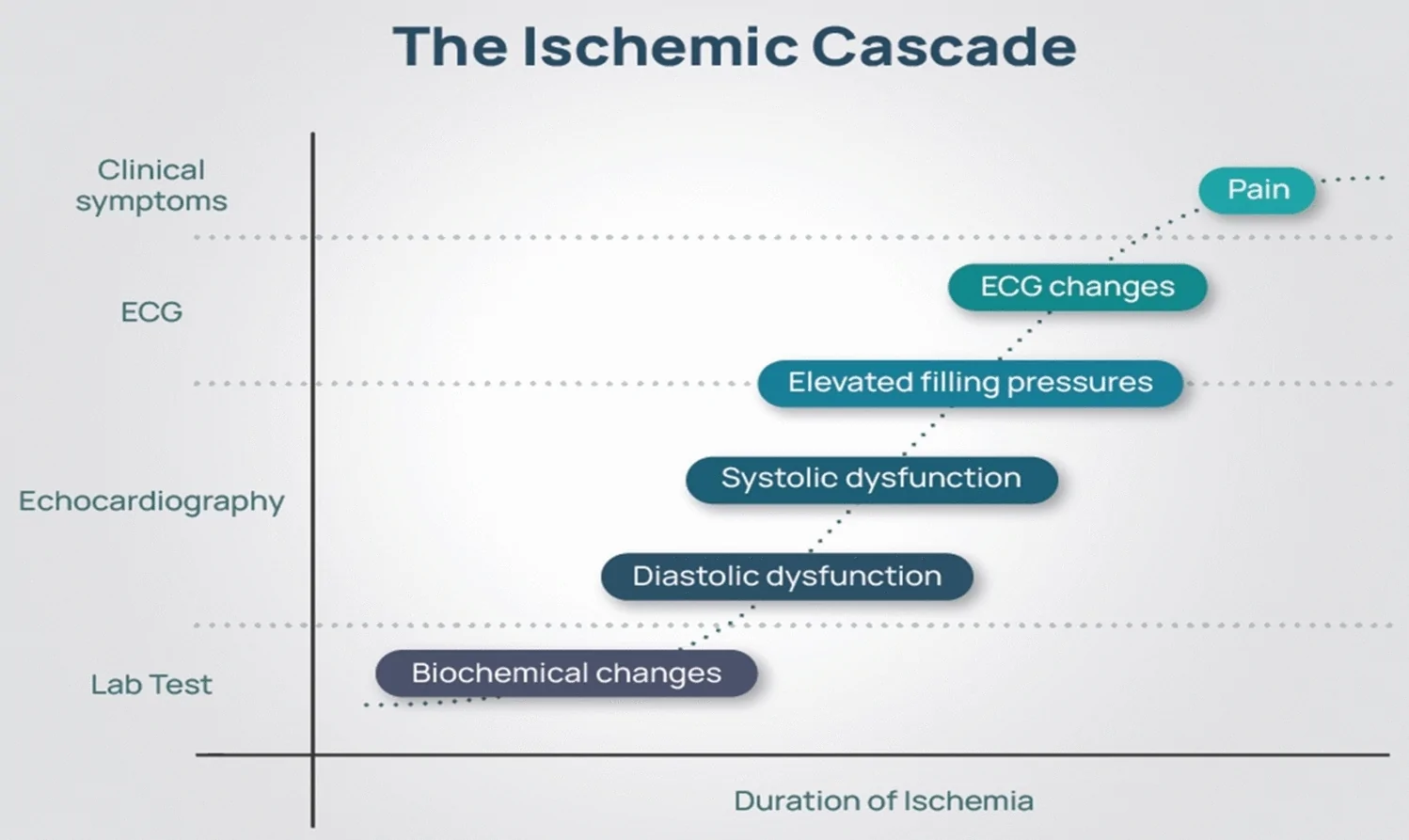
The ischemic cascade

The rationale for performing early echocardiography in patients with chest pain is traditionally based on the pathophysiology of the ischaemic cascade [21] (Fig. 5). Interestingly, RWA was uncommon in patients with acute coronary syndrome. However, when such impairment was detected, it was highly predictive of coronary artery occlusion. The long-standing classification of acute coronary syndromes into STEMI and non-STEMI, which was used in the study, is gradually being replaced by a new classification system that distinguishes between occlusive myocardial infarction (OMI) and non-occlusive myocardial infarction (NOMI) [22]. This paradigm shift is based on evidence that a significant proportion of non-STEMI patients have occlusive coronary lesions and would therefore benefit from early coronary angiography [23]. It corresponds well with our finding that patients with difficult-to-interpret findings (e.g. bundle branch block, ST elevations not meeting STEMI criteria) benefited most from POCE. We hypothesise that the detection of RWA by POCE of sufficient quality is superior to ECG for identifying high-risk patients and we advocate initiating research in this area. Nevertheless, it is important to emphasise that the standard troponin algorithm remains the primary method for ruling out acute coronary syndrome in most patients presenting with acute chest pain.

Archiving and sharing POCE records has been shown to be crucial in facilitating collaboration with consulting specialists, especially when RWA was present. We therefore strongly support the European Association of Cardiovascular Imaging’s recommendation to archive POCE examinations [24].

Patients without ACS (such as those with severe valvular disease or pulmonary embolism) experienced a shorter Emergency Department stay. In these cases, POCE utilisation facilitated rapid diagnosis. Such patients also avoid time-consuming outpatient management, which may include coronary angiography. This is another main contributor to reducing the time to coronary angiography. Early recognition of less common causes of pain was also the main reason for the trend towards a higher number of hospital admissions in the POCE group.

Although the training in this study was based on the BSE Level 1 protocol, which is considered to be one of the most valuable focused protocols[25] and demonstrated very good clinical application in this study, physicians often expanded the scope of the examination to reliably detect regional wall motion abnormalities. This finding is consistent with previous findings that assessment of wall motion abnormalities is poorly predictive of acute coronary syndrome when insufficiently detailed POCE protocols are used [26]. We conclude that BSE Level 1 protocol is a good basis for routine use in clinical practice. However, we anticipate that a protocol specifically designed for efficient examining patients with acute chest pain will be introduced in the future.

We also recommend the development of evidence-based international recommendations for POCE training for basic clinical syndromes, which may broaden the impact of POCE and consolidate its role in emergency medicine [15]. The training programme described in this article is demanding and may not be feasible in all centres. However, we believe that our data could help healthcare authorities and facility managers to make evidence-based decisions about the cost-effective training of non-cardiologists in POCE. In addition, we support the American Society of Echocardiography’s recommendation that cardiologists should lead the training of non-cardiologists in echocardiography, with an emphasis on standardised, competency-based service delivery [6, 27–29].

### Study limitations

The main limitation of this study is its monocentric design. We expect that the benefits of POCE may differ from those observed in tertiary cardiovascular centres. Acceleration of chest pain management may not be feasible in centres that are unable to respond immediately to the full range of POCE findings, either due to lack of resources or free capacity. The benefit may also be compromised by mistrust of other specialists in the reliability of POCE performed by emergency physicians.

The study was not designed to prove improved diagnostic accuracy by POCE. This is because, at the time the study was designed, there was more concern about the high error rate of POCE carried out by non-cardiologists. For this reason, the error rate assessment was only included as a safety endpoint.

The randomisation process cannot be blinded due to the nature of POCE, which requires direct interaction between the clinician and the process of performing the ultrasound examination. The chosen study design does not distinguish to what extent the disclosure of key findings or the increase in physician confidence in the diagnostic process is the cause of the acceleration of care.

Patients for whom POCE could not be performed within 90 min of arriving at the ED were excluded from the study. The number of such patients in the study population was very low. In chronically congested EDs, this condition may significantly limit the generalisability of the results. The number of patients included does not allow reliable subanalysis for rare diseases and ECG findings. Given the monocentric nature of the study, the impact of POCE on the management of less common conditions may also be biased.

Another limitation of the study is the small number of physicians performing POCE examinations. Without enthusiasm for the implementation of POCE in clinical practice and confidence in the method, the benefits may be reduced or absent.

## Conclusion

The results of our study clearly demonstrate the significant benefits of POCE performed in the ED by non-cardiologists who have undergone structured training. In particular, the implementation of POCE led to a significant reduction in length of ED stay and decision-making times, which are critical measures of the efficiency of emergency care. The time to coronary angiography was also reduced. The accuracy of initial diagnosis improved significantly, highlighting the clinical value of POCE in the management of acute chest pain.

Where a standardised and comprehensive POCE education is required, the British Society of Echocardiography’s Level 1 training framework represents an appropriate foundational protocol.

## Supplementary Information

Below is the link to the electronic supplementary material.

Supplementary file1 (DOCX 22 KB)

Supplementary file2 (DOCX 21 KB)

## Data Availability

The data are available from the corresponding author upon reasonable request.
