# Supplementary material for: Effect of point-of-care echocardiography by noncardiologists on patient management in acute chest pain
Source: Intern Emerg Med. 2025 Nov 24;21(8):3025–35. doi: 10.1007/s11739-025-04198-6 (PMC13751739; doi:10.1007/s11739-025-04198-6)
Supplement: Supplementary file 1 [file 11739_2025_4198_MOESM1_ESM.docx]

Supplementum 1: FOCUS logbook

**NAME:**

| **Finding** | **Date** | **Workplace** | **Supervisor** | **Signature** |
| --- | --- | --- | --- | --- |
| dilatation/dysfunction of left ventricle |  |  |  |  |
| dilatation/dysfunction of left ventricle |  |  |  |  |
| dilatation/dysfunction of left ventricle |  |  |  |  |
| dilatation/dysfunction of left ventricle |  |  |  |  |
| dilatation/dysfunction of left ventricle |  |  |  |  |
| dilatation/dysfunction of right ventricle |  |  |  |  |
| dilatation/dysfunction of right ventricle |  |  |  |  |
| dilatation/dysfunction of right ventricle |  |  |  |  |
| dilatation/dysfunction of right ventricle |  |  |  |  |
| pericardial effusion |  |  |  |  |
| pericardial effusion |  |  |  |  |
| tamponade |  |  |  |  |
| hypovolemia |  |  |  |  |
| hypovolemia |  |  |  |  |
| hypovolemia |  |  |  |  |
| CPR |  |  |  |  |
| CPR |  |  |  |  |
| Pleural effusion |  |  |  |  |
| Pleural effusion |  |  |  |  |
| B- lines |  |  |  |  |
| B - lines |  |  |  |  |
| Aortic valve stenosis |  |  |  |  |
| Aortic valve stenosis |  |  |  |  |
| Aortic valve insuff. |  |  |  |  |
| Mitral valve insuff. |  |  |  |  |
| Mitral valve insuff. |  |  |  |  |
| Tric. valve insuff./ pulm. hypertension |  |  |  |  |
| Tric. valve insuff./ pulm. hypertension |  |  |  |  |
| Aortic root dilatation |  |  |  |  |

**Echo simulator**

| **day:** | **Time** |
| --- | --- |
|  |  |
|  |  |
|  |  |
|  |  |
|  |  |
|  |  |
