# Supplementary material for: Effect of point-of-care echocardiography by noncardiologists on patient management in acute chest pain
Source: Intern Emerg Med. 2025 Nov 24;21(8):3025–35. doi: 10.1007/s11739-025-04198-6 (PMC13751739; doi:10.1007/s11739-025-04198-6)
Supplement: Supplementary file 2 [file 11739_2025_4198_MOESM2_ESM.docx]

|  | Bundle branch block | | ST elevation  (not fulfilling STEMI criteria) | | ST-depression | | T-wave abnormity | | No acute QRS-T abnormity | | |
| --- | --- | --- | --- | --- | --- | --- | --- | --- | --- | --- | --- |
|  | POCE (n=10) | Control (n=6) | POCE (n=6) | Control (n=3) | POCE (n=12) | Control (n=7) | POCE (n=7) | Control (n=8) | POCE (n=39) | Control (n=52) |  |
| Length of ED  stay [hours] | 2:40:18  ± 1:39:02 | 5:58:40  ± 0:50:46 | 2:56:43  ± 1:08:49 | 6:05:00  ± 2:27:00 | 3:07:30  ± 1:21:09 | 3:30:17  ± 1:44:54 | 3:58:09  ± 1:33:18 | 4:05:00 ± 1:30:10 | 3:56:03  ± 1:40:21 | 4:36:14 ± 1:41:45 |  |
| Time to decision [hours] | 2:18:06  ± 1:41:00 | 5:02:50  ± 0:51:17 | 1:51:34  ± 1:04:50 | 5:45:00  ± 2:32:00 | 2:35:50  ± 1:31:08 | 3:16:09  ± 1:41:44 | 3:27:00 ± 1:33:41 | 3:30:00 ± 1:36:21 | 2:51:35 ± 2:02:28 | 4:07:30 ± 1:33:26 |  |

Supplementum 2: Difference in length of ED stay and time to decision according to the presenting ECG.

ED – Emergency Department

POCE – Poit of care echocardiography

Supplementum 3: Difference in length of ED stay and time to decision according to the final diagnosis (only findings with n=2 or more are included).

|  | Acute coronary syndrome | | Non-cardiac pain* | | Pericarditis** | | Aortic stenosis | |
| --- | --- | --- | --- | --- | --- | --- | --- | --- |
|  | POCE (n=13) | Control (n=5) | POCE (n=50) | Control (n=66) | POCE (n=3) | Control (n= 2) | POCE (n=2) | Control (n=2) |
| Length of ED  stay [hours] | 2:43:32  ± 1:41:28 | 2:26:00 ± 0:57:36 | 4:01:04 ± 1:30:23 | 4:41:19 ± 1:41:48 | 3:59:00 ± 1:01:00 | 5:51:00 ± 1:04:00 | 0:52:30 ± 0:02:30 | 4:38:30 ± 2:34:30 |
| Time to decision [hours] | 2:08:51 ± 1:43:41 | 2:04:15 ± 0:47:03 | 3:05:06 ± 1:52:42 | 4:10:58 ± 1:35:23 | 3:34:00 ± 1:26:00 | 5:46:00 ± 1:09:00 | 0:28:30  ± 0:03:30 | 3:54:30 ± 1:58:30 |

*Serial troponine negative and HEART score low or dobutamine stress echo / coronary angiography negative

** At least two criteria according to the 2015 ESC guidelines were present.

ED – Emergency Department

POCE – Poit of care echocardiography
